# Soccer and Benign Paroxysmal Positional Vertigo

**DOI:** 10.1155/2023/3744863

**Published:** 2023-02-14

**Authors:** Nikolaj Warming, Stephanie Balslev Andersen, Dan Dupont Hougaard

**Affiliations:** ^1^Department of Clinical Medicine, Aalborg University, Aalborg, Denmark; ^2^Balance & Dizziness Centre, Department of Otolaryngology, Head & Neck Surgery and Audiology, Aalborg University Hospital, Aalborg, Denmark

## Abstract

*Introduction*. Benign paroxysmal positional vertigo (BPPV) is the most common cause of vertigo among adults. The etiology of BPPV is unknown in approximately 50 percent of cases. This condition is also termed primary BPPV, if the etiology is unknown, and secondary BPPV if patients have identified predisposing factors. A few studies suggest that there is a correlation between the development of BPPV and specific sports. *Case Report*. A 19-year-old male presented with recurrent episodes of vertigo during soccer play. Eight months prior to referral, the patient was involved in a car accident with a mild head trauma. The patient was later diagnosed with BPPV several times. *Discussion*. Soccer might be a plausible BPPV trigger, especially if there is a prehistory of head trauma. This is most likely due to the demands of the game such as the change of directions, repetitive head impacts (headers or head collisions), accelerations/decelerations, jumps, foot landings, and rapid head movements.

## 1. Introduction

Benign paroxysmal positional vertigo (BPPV) is a peripheral vestibular disorder that is considered the most common cause of vertigo among adults. The cumulative incidence is 10 percent within the general population. Any of the six semicircular canals (SCCs) may be affected, but most frequently, otoconias are displaced into the posterior SCC (80–90 percent). The second most common location is the lateral SCC (5–30 percent). The anterior SCC is infrequently affected and represents only 1-2 percent of all BPPV cases. The prevalence of bilateral affection of the posterior SCCs is higher in posttraumatic than idiopathic cases [1, 2].

The etiology of BPPV is unknown in approximately 50 percent of cases. This condition is also termed primary BPPV. The remaining cases include secondary BPPV with known predisposing factors such as other peripheral vestibular disorders such as temporal bone fractures, Ménière's disease, vestibular neuritis, stapedectomy, and other types of ear surgery, viral infections, inner ear infections, hypertension, and long-term immobilization [2, 3]. A few previous studies suggest that there is a correlation between the development of BPPV and specific sports, such as American football, soccer, swimming, and mountain biking [2–6].

There are two known subtypes of BPPV: (1) canalolithiasis (CAN) that describes the condition with free floating otoconia within the SCCs and (2) cupulolithiasis (CUP) that describes the condition where otoconia are attached to the cupula within the SCCs [1].

Overall, the Bárány Society as well as the AAO-HNS diagnostic BPPV criteria are used as the predominant criteria for placing a diagnosis of BPPV at our tertiary University Hospital Balance & Dizziness Centre. However, when considering the literature dealing with the diagnosis of posterior and lateral CAN, some authors suggest further subdivision into two types: (1) ampullary-arm CAN and (2) non-ampullary-arm CAN [1, 7, 8].

The otolithic organs, within the inner ear, contain otoconias which are embedded in a gelatinous mass. With CAN, these small calcium-carbonate crystals dislodge from the utricle and enter one or several of the SCCs. The dislodged otoconia alter the properties of the endolymph by displacement of the hair cells within the cupula, causing a sensation of concomitant rotational movement (vertigo).

With CUP, the otoconia adhere to the cupula within the SCC [1].

BPPV is characterized by brief, transient episodes of vertigo triggered by specific head positions that correspond to movements in the planes of the affected SCCs. BPPV symptomology includes recurrent attacks of rotational vertigo, which predominantly last 10–30 seconds. Concomitant vegetative symptoms include paleness, nausea, vomiting, tachycardia, and excessive sweating [1, 5].

With CAN, the duration of the attacks is less than one minute and with CUP, the symptoms persist for more than one minute [1].

To examine the individual SCCs, different positional tests are used. The Dix–Hallpike (DH) test examines the vertical SCCs by turning the head of a sitting patient 45 degrees to either side and moving the patient backwards in the supine position with the head extended 20–30 degrees. The supine roll test (SRT) examines the lateral SCCs by quickly turning the patient's head from side-to-side in the supine position with the head flexed 30 degrees [1].

Objective findings are as follows:

CAN: Recurrent attacks of positional vertigo. The duration of the individual attack is *less* than 60 seconds. Positional nystagmus (PN) is observed with a *short latency* of 2–5 seconds. Fatigability is seen with repetitive positional testing.

Nystagmus characteristics are as follows:

(i) Posterior CAN:
  - *Intermittent* vertical upbeat PN with a concomitant torsional component beating towards the affected side with DH-testing.
(ii) Lateral CAN:
  - *Long lasting* bilateral geotropic PN (most pronounced on the affected side) with SRT-testing.

CUP: Recurrent attacks of positional vertigo. The duration of individual attacks is *more* than 60 seconds. The duration can be longer if the head is kept in the provoking position. *No or brief latency* of PN. Non fatigable with repetitive testing.

Nystagmus characteristics:

- Posterior CUP: *Persisting* vertical upbeat PN with a concomitant torsional component beating towards the affected side with DH-testing.
- Lateral CUP: *Persisting* bilateral apogeotropic PN (most pronounced on the nonaffected side) with SRT-testing [1].

Treatment of BPPV is done by specific repositioning maneuvers that are targeting the affected SCCs [9]. Complete resolution of BPPV one month after successful treatment is seen with 20–80% of cases [10]. Recurrence of vertigo (and BPPV) is quite common with both posttraumatic and idiopathic BPPV. In follow-up trials, one-year recurrence rates of 15 percent and five-year recurrence rates of 37–50 percent have been reported. Posttraumatic BPPV seems to have higher recurrence rates than idiopathic BPPV. A common complication with BPPV treatment is ipsilateral SCC conversion from one SCC to another [10].

## 2. Case

A 19-year-old male was referred from the neurological department to the tertiary Balance & Dizziness Centre at the Department of Otolaryngology, Head & Neck Surgery at Aalborg University Hospital, Aalborg, Denmark, for assessment of recurrent episodes of vertigo during soccer play. Prior to the referral, a thorough neurological examination concluded that no abnormal objective neurological findings were present except for objective findings of an uncharacteristic and unspecified nystagmus together with subjective complaints of vertigo.

A cerebral MRI revealed structural changes within the right middle cerebellar peduncle which also included part of the right cerebellar hemisphere. Within the right cerebellar hemisphere, primarily in the white matter and in the middle cerebellar peduncle, a poorly defined, almost homogeneous structure measuring approximately 2.7 × 1.7 centimeters was found. There was no significant mass effect, diffusion inhibition, or bleeding sequelae. Following intravenous contrast administration, no contrast loading was observed. The changes were hypointense on T1-weighted sequences and hyperintense on T2- and FLAIR-weighted sequences. The patient underwent consecutive MRI scans with one-year intervals during an eight-year period from 2015 until 2022. No changes in size and/or morphology were observed throughout the entire observation period. The findings were concluded to be either postinflammatory or postinfectious changes.

Eight months prior to the referral, the patient was involved in a car accident with a mild head trauma. The following months, the patient experienced intermittent vertigo triggered by repetitive alternating fast head movements, especially during soccer practice and sometimes during forward bends. The vertiginous episodes lasted for minutes to a few hours before the patient experienced complete remission of symptoms. No concomitant symptoms such as nausea, vomiting, pain, tinnitus, or hearing impairment were reported. Teammates, however, had observed the paleness of the patient's face. The patient had no prior medical history of any otological or neurological disease and did not take any prescription medication.

The following two years, ten examinations and treatment sessions were scheduled (refer to Table 1: positive findings and diagnostics). Pathological objective findings were sparse except for intermittent positional and spontaneous nystagmus and concomitant subjective complaints of vertigo. The vestibular function was examined by video head impulse testing (vHIT). This examination showed normal vestibulo-ocular-reflex (VOR) function of all six semicircular canals (SCCs) with mean gain values within the normative range and no pathological saccades present. Furthermore, a mechanical rotational chair (TRV-chair®, Interacoustics©, Middelfart, Denmark) was used for diagnostics and treatment of benign paroxysmal positional vertigo (BPPV) by means of the DH- and the SRT tests. The patient was diagnosed with CUP subtype BPPV at several follow-ups, but was located within different SCCs bilaterally. The left lateral and the right posterior SCCs were affected at some point throughout the two-yearfollow-ups.

A final follow-up examination was performed six years after the initial referral. This follow-up examination concluded that the patient had a complete normal otoneurological examination and had neither complaints nor symptoms compatible with BPPV since his ninth visit (five years prior to this follow-up).

**Figure 1:**
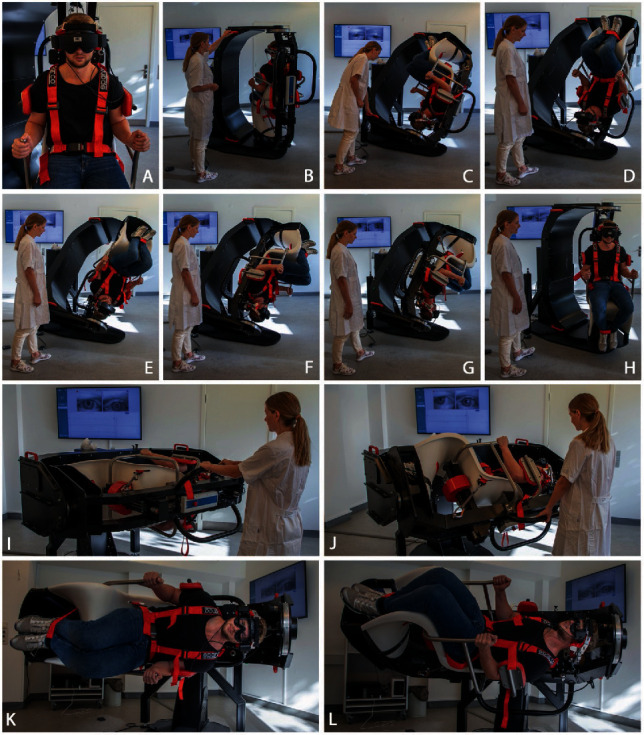
Treatment with the TRV-chair. Potentiated Epley's maneuver (a–h) and the dynamic barbecue roll maneuver (i–l). (a) Patient positioned in the TRV-chair with VNG Goggles in place (vision denied). (b) The patient is placed upright and turned 45 degrees to the left in the yaw axis as part of the initial position for a left Dix–Hallpike test. (c) The patient is moved backwards into a position corresponding to the left Dix–Hallpike test position. (d–g) The patient is continuously rotated 45 degree towards the healthy ear following treatment in every position. (c–g) Please note that 10 shocks were applied in every of these five positions to add kinetic energy during repositioning. (h) The patient is reverted to the upright position. (i) Starting position of the dynamic barbecue roll maneuver for treatment of right horizontal cupulolithiasis. (j–l) The patient is rotated 360 degrees ten times towards the healthy ear with a combination of accelerations and decelerations in the roll axis (in this case treatment of right sided lateral cupulolithiasis).

The treatments given with the TRV-chair included the dynamic barbeque roll maneuver (treatment of lateral BPPV) and the potentiated Epley maneuver (treatment of posterior BPPV), where repositioning with concomitant kinetic forces were applied. For details, please refer to Figure 1.

Following ten treatment sessions in total, all objective vestibular tests were normal at the final follow-up examination, and the patient was relieved of all vertiginous symptoms related to activities of daily life and soccer practice. Informed consent from the patient, for publication of this case report, was obtained prior to the submission of this case report.

## 3. Discussion

No previous studies have identified a direct correlation between soccer play and the triggering of BPPV. A study by Kerrigan et al. [11] describes that 22 percent of young adults diagnosed with BPPV had a history of amateur soccer play, suggesting a possible association between BPPV and soccer. A previous case report described an elite soccer player with BPPV. This case of BPPV occurred during a change of position from supine to an upright sitting position with rapid head movements [3].

Similarities between the elite soccer player and the patient in this case report consisted of a prehistory of head trauma and soccer play and vertigo triggered by rapid head movements. However, there are also some differences between the two cases. The patient in this case primarily experienced vertigo during soccer play, and his BPPV condition was more resistant to repositioning treatment.

The elite soccer player, who was diagnosed with left posterior CAN, was treated and cured by means of the standard Epley maneuver and experienced no relapse.

The patient in this study was primarily diagnosed with CUP of the left lateral SCC. The patient was treated several times with repositioning maneuvers with the TRV-chair. However, the BPPV in this case was very resistant to treatment. This further supports the diagnosis of CUP. Some might argue that these findings could also be caused by ampullary-arm BPPV of the lateral SCC, causing an apogeotropic form of BPPV [12]. With posttraumatic BPPV, the subtype is often CUP and the location bilateral. This might explain why secondary BPPV, caused by head trauma, may require several treatment sessions and is more retractable compared to “standard idiopathic BPPV cases.”

One study suggests that 11-12 percent of cases with PN are of central origin [13]. As this patient also had atypical findings within the cerebellum following MRI examinations, a central origin/trigger of the observed PN cannot be ruled out completely. Consecutive MRI follow-up scans, however, showed no further development of the findings within the cerebellum. At two follow-up visits, the patient had discrete and very low-velocity spontaneous nystagmus without any torsional components (visits 2 and 6). The patient was tested with visual fixation at both visits. At the second visit, fixation led to complete remission of the spontaneous nystagmus, while the effect of fixation was not described at the sixth visit. Canalith jam in the posterior SCC should be considered in cases of spontaneous downbeating nystagmus. A complete canalith jam leads to a blockage in endolymphatic flow and a persistent ampullopetal displacement of the posterior canal cupula, thus leading to a spontaneous downbeating nystagmus regardless of head position [14–16]. Another possible cause of downbeating nystagmus could include the apogeotropic form of posterior BPPV [8].

Positional nystagmus can be related to a central or a peripheral pathology. The reported vertigo and the concomitantly observed PN were in accordance with the characteristic BPPV-related symptoms as well as the BPPV-related PN. Additionally, both the PN observed by the examiner as well as the vertiginous symptoms reported by the patient completely disappeared following several treatments. All of the abovementioned observations/reporting indicate a peripheral pathology in relation to this case report. If the vertigo and PN were caused by central pathology, one would not expect complete remission of both subjective symptoms and objective findings following targeted repositional maneuvers.

One study suggests that there might be an increased risk of BPPV with soccer play due to the demands of the game such as changes of direction, repetitive headers, collisions, accelerations/decelerations, jumps, foot landings, and rapid head movements [3].

BPPV is also described in relation to other sports activities. One study determined that there was a correlation between mountain biking and the development of BPPV. Accelerations and decelerations, shocks, and vibrations during mountain bike riding were described as possible BPPV triggers [4].

Another study found a possible correlation between rapid head movements with swimming and the development of BPPV. The rapid head movements during swimming might cause otoconia to be dislodged from the utricular macula into any of the SCCs. The subject in that case report, however, had no prior history of head injuries. This indicates that rapid head movement alone might also act as a BPPV trigger. The rapid head movements can be compared to some of the movements during soccer play, especially during changes of direction. The authors of this study did not find any correlation between the development of BPPV and either the frequency or duration of training sessions, but they did find a possible correlation with the intensity of the training [5, 6].

A third study, with American football players and BPPV, showed otherwise. There was a clear correlation between both the frequency and the duration of the training sessions and the development of BPPV. This may be explained by the number of collisions as well as the frequency of head traumas [2]. It is important to distinguish between PN that appears in relation to a concussion (brain injury) and the PN that is characteristic for BPPV. The main differences between these two types of PN, besides the case history, are the absence of a crescendo-decrescendo aspect with a brain injury, as well as the fact that with a brain injury it may be a centrally induced PN that typically does not include the mixed components of PN (e.g., a combination of horizontal/vertical and torsional PN), but more often just purely horizontal or pure vertical eye movements [17]. In addition to this, other oculomotor abnormalities like smooth pursuit, saccade, and gaze abnormalities may be seen with traumatic brain injuries, and this is equally important to consider whenever patients with head injuries are evaluated, e.g., by means of VideoNystagmoGraphy (VNG) examination [18].

Otorhinolaryngologists and sports physicians should be aware of this sports-related (posttraumatic) type of BPPV. In some cases, BPPV might be mistaken for a concussion. Treatments and recovery time vary greatly among these two conditions. Fast and precise diagnostics and treatment of BPPV among soccer players seem important in order to achieve a fast recovery and a timely return to sports activities. Furthermore, it is noticeable that the patient was diagnosed and treated using a TRV-chair with superior diagnostics and several treatment options. It might not have been possible to either diagnose or treat this patient with a traditional positional tests and repositional maneuvers possible with a traditional examination bed.

## 4. Conclusion

Soccer play might be a plausible BPPV trigger, especially if there is a prehistory of head trauma. This is most likely due to the demands of the game such as changes of direction, repetitive headers, collisions, accelerations/decelerations, jumps, foot landings, and rapid head movements. Additional research related to soccer play is recommended in order to assess and determine the impact of soccer play on the risk of developing BPPV.

## Figures and Tables

**Table 1:** Objective findings related to diagnostics. ^*∗*^Follow-up examination six years after the first examination. Please note that, during the symptomatic period, the patient was diagnosed with BPPV at 5 out of 8 (63%) follow-up visits. Please also note that diagnostic conclusions in this table are based exclusively on the Bárány- and AAO-HNS diagnostic criteria. Other possible types of BPPV are mentioned in the discussion section. Discrete spontaneous nystagmus is equivalent to an average slow phase velocity (a-SPV) of less than 2 degrees per second.

Visit	Positive objective findings	Diagnostics in relation to BPPV
1	SRT: persisting bilateral apogeotropic nystagmus (>1 minute), highest slow phase velocity on the right side	CUP of the left lateral SCC
2	SRT: persisting bilateral apogeotropic nystagmus (>1 minute), highest slow phase velocity on the right side Discrete right-beating horizontal spontaneous nystagmus	CUP of the left lateral SCC
3	Right DH: persisting upbeat and rotational nystagmus (>1 minute) SRT: persisting bilateral apogeotropic nystagmus (>1 minute), highest slow phase velocity on the right side	CUP of the right posterior SCC and CUP in the left lateral SCC
4	Right DH: persisting upbeat and rotational nystagmus (>1 minute) SRT: persisting bilateral apogeotropic nystagmus (>1 minute), highest slow phase velocity on the right side	CUP of the right posterior SCC and CUP of the left lateral SCC
5	Normal otoneurological examination	Patient symptomatic, but no objective findings compatible with BPPV
6	Discrete downbeating vertical spontaneous nystagmus	Patient symptomatic, but no objective findings compatible with BPPV
7	Normal otoneurological examination	Patient symptomatic, but no objective findings compatible with BPPV
8	SRT: persisting bilateral apogeotropic nystagmus (>1 minute), highest slow phase velocity on the right side	CUP of the left lateral SCC
9	Normal otoneurological examination	No objective findings or symptoms compatible with BPPV
10	Normal otoneurological examination	No objective findings or symptoms compatible with BPPV
11^*∗*^	Normal otoneurological examination	No objective findings or symptoms compatible with BPPV
